# 2,4-Dimethyl-*N*-(3-methyl­phen­yl)benzene­sulfonamide

**DOI:** 10.1107/S1600536810011529

**Published:** 2010-03-31

**Authors:** P. G. Nirmala, B. Thimme Gowda, Sabine Foro, Hartmut Fuess

**Affiliations:** a Department of Chemistry, Mangalore University, Mangalagangotri 574 199, Mangalore, India; b Institute of Materials Science, Darmstadt University of Technology, Petersenstrasse 23, D-64287 Darmstadt, Germany

## Abstract

In the structure of the title compound, C_15_H_17_NO_2_S, the dihedral angle between the two aromatic rings is 47.1 (1)°. In the crystal structure, mol­ecules are connected by N—H⋯O hydrogen bonds, forming *C*(4) chains running along the *c* axis.

## Related literature

For the preparation of the title compound, see: Savitha & Gowda (2006 ▶). For our studies of the effect of substituents on the structures of *N*-(ar­yl)aryl­sulfonamides, see: Gowda *et al.* (2009 ▶, 2010 ▶); Nirmala *et al.* (2009 ▶). For related structures, see: Gelbrich *et al.* (2007 ▶); Perlovich *et al.* (2006 ▶).
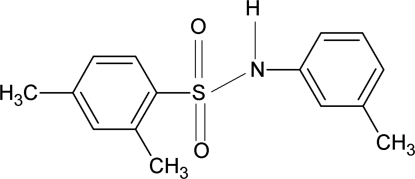


## Experimental

### 

#### Crystal data

C_15_H_17_NO_2_S
*M* *_r_* = 275.36
Monoclinic, 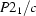

*a* = 9.110 (1) Å
*b* = 15.367 (2) Å
*c* = 10.422 (1) Å
β = 104.05 (1)°
*V* = 1415.4 (3) Å^3^
*Z* = 4
Mo *K*α radiation
μ = 0.23 mm^−1^
*T* = 299 K
0.40 × 0.40 × 0.26 mm

#### Data collection

Oxford Diffraction Xcalibur diffractometer with a Sapphire CCD detector
Absorption correction: multi-scan (*CrysAlis RED*; Oxford Diffraction, 2009 ▶) *T*_min_ = 0.915, *T*_max_ = 0.944
5538 measured reflections
2870 independent reflections
2375 reflections with *I* > 2σ(*I*)
*R*_int_ = 0.012

#### Refinement

*R*[*F* ^2^ > 2σ(*F* ^2^)] = 0.040
*wR*(*F* ^2^) = 0.112
*S* = 1.06
2870 reflections
178 parameters
H atoms treated by a mixture of independent and constrained refinement
Δρ_max_ = 0.23 e Å^−3^
Δρ_min_ = −0.34 e Å^−3^

### 

Data collection: *CrysAlis CCD* (Oxford Diffraction, 2009 ▶); cell refinement: *CrysAlis RED* (Oxford Diffraction, 2009 ▶); data reduction: *CrysAlis RED*; program(s) used to solve structure: *SHELXS97* (Sheldrick, 2008 ▶); program(s) used to refine structure: *SHELXL97* (Sheldrick, 2008 ▶); molecular graphics: *PLATON* (Spek, 2009 ▶); software used to prepare material for publication: *SHELXL97*.

## Supplementary Material

Crystal structure: contains datablocks I, global. DOI: 10.1107/S1600536810011529/bt5222sup1.cif

Structure factors: contains datablocks I. DOI: 10.1107/S1600536810011529/bt5222Isup2.hkl

Additional supplementary materials:  crystallographic information; 3D view; checkCIF report

## Figures and Tables

**Table 1:** Hydrogen-bond geometry (Å, °)

*D*—H⋯*A*	*D*—H	H⋯*A*	*D*⋯*A*	*D*—H⋯*A*
N1—H1*N*⋯O2^i^	0.83 (2)	2.13 (3)	2.932 (2)	162 (2)

Symmetry code: (i) 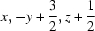.

**Fig. 1.**
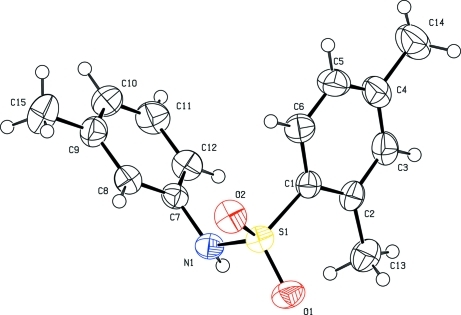
Molecular structure of the title compound, showing the atom labelling scheme. Displacement ellipsoids are drawn at the 50% probability level.

**Fig. 2.**
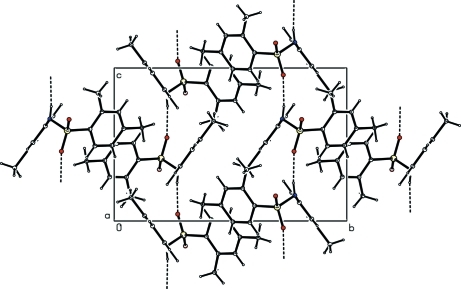
Molecular packing of the title compound with hydrogen bonding shown as dashed lines.

## supplementary crystallographic information

### Comment

As part of a study of the effect of substitutions on the structures of *N*-(aryl)-arylsulfonamides (Gowda *et al.*, 2009; Gowda *et al.*, 2010; Nirmala *et al.*, 2009), in the present work, the structure of 2,4-dimethyl-*N*-(3-methylphenyl)benzenesulfonamide (I) has been determined (Fig. 1). The conformation of the N—C bond in the C—SO_2_—NH—C segment has *gauche* torsions with respect to the S═O bonds. The molecule is twisted at the S—N bond with the C1—SO_2_—NH—C7 torsion angle of -58.4 (2)°, compared to the values of 71.6 (1)° in 2,4-dimethyl-*N*-(3-methylphenyl)benzenesulfonamide (II)(Nirmala *et al.*, 2009), -46.1 (3)° (molecule 1) & 47.7 (3)° (molecule 2) in the two molecules of 2,4-dimethyl-*N*-(phenyl)- benzenesulfonamide (III)(Gowda *et al.*, 2009) and 55.8 (2)° and -58.4 (3)°, respectively, in the 2 molecules of *N*- (3-methylphenyl)benzenesulfonamide (IV)(Gowda *et al.*, 2010). The conformation of the N—H bond in (I) is *anti* to the 3-methyl group in the aniline benzene ring, compared the *syn* conformation observed between the N—H bond and the 3-methyl group in (IV).

The sulfonyl benzene and the aniline benzene rings in (I) are tilted relative to each other by 47.1 (1)°, compared to the values of 47.0 (1)° in (II), 67.5 (1)° in molecule 1 and 72.9 (1)° in molecule 2 of (III), and 67.9 (1)° in molecule 1 and 68.6 (1)° in molecule 2 of (IV).

The other bond parameters in (I) are similar to those observed in (II), (III), (IV) and other aryl sulfonamides (Perlovich *et al.*, 2006; Gelbrich *et al.*, 2007). The crystal packing shows that the molecules are connected by N—H···O hydrogen bonds (Table 1) to chains running along the c axis (Fig. 2).

### Experimental

The solution of 1,3-xylene (1,3-dimethylbenzene) (10 ml) in chloroform (40 ml) was treated dropwise with chlorosulfonic acid (25 ml) at 0 ° C. After the initial evolution of hydrogen chloride subsided, the reaction mixture was brought to room temperature and poured into crushed ice in a beaker. The chloroform layer was separated, washed with cold water and allowed to evaporate slowly. The residual 2,4-dimethylbenzenesulfonylchloride was treated with *m*-toluidine in the stoichiometric ratio and boiled for ten minutes. The reaction mixture was then cooled to room temperature and added to ice cold water (100 ml). The resultant solid 2,4-dimethyl-*N*-(3-methylphenyl)benzenesulfonamide was filtered under suction and washed thoroughly with cold water. It was then recrystallized to constant melting point from dilute ethanol. The purity of the compound was checked and characterized by recording its infrared and NMR spectra (Savitha & Gowda, 2006). The prism like colourless single crystals used in X-ray diffraction studies were grown in ethanolic solution by a slow evaporation at room temperature.

### Refinement

The H atom of the NH group was located in a difference map and its positional parameters were refined. The other H atoms were positioned with idealized geometry using a riding model with C—H = 0.93–0.96 Å. All H atoms were refined with isotropic displacement parameters set to 1.2 times of the *U*_eq_ of the parent atom.

### Crystal data

**Table:**

C_15_H_17_NO_2_S	*F*(000) = 584
*M**_r_* = 275.36	*D*_x_ = 1.292 Mg m^−^^3^
Monoclinic, *P*2_1_/*c*	Mo *K*α radiation, λ = 0.71073 Å
Hall symbol: -P 2ybc	Cell parameters from 3146 reflections
*a* = 9.110 (1) Å	θ = 2.6–27.7°
*b* = 15.367 (2) Å	µ = 0.23 mm^−^^1^
*c* = 10.422 (1) Å	*T* = 299 K
β = 104.05 (1)°	Prism, colourless
*V* = 1415.4 (3) Å^3^	0.40 × 0.40 × 0.26 mm
*Z* = 4	

### Data collection

**Table:**

Oxford Diffraction Xcalibur diffractometer with a Sapphire CCD detector	2870 independent reflections
Radiation source: fine-focus sealed tube	2375 reflections with *I* > 2σ(*I*)
graphite	*R*_int_ = 0.012
Rotation method data acquisition using ω and phi scans	θ_max_ = 26.4°, θ_min_ = 2.7°
Absorption correction: multi-scan (*CrysAlis RED*; Oxford Diffraction, 2009)	*h* = −11→9
*T*_min_ = 0.915, *T*_max_ = 0.944	*k* = −19→14
5538 measured reflections	*l* = −13→10

### Refinement

**Table:**

Refinement on *F*^2^	Primary atom site location: structure-invariant direct methods
Least-squares matrix: full	Secondary atom site location: difference Fourier map
*R*[*F*^2^ > 2σ(*F*^2^)] = 0.040	Hydrogen site location: inferred from neighbouring sites
*wR*(*F*^2^) = 0.112	H atoms treated by a mixture of independent and constrained refinement
*S* = 1.06	*w* = 1/[σ^2^(*F*_o_^2^) + (0.0485*P*)^2^ + 0.8232*P*] where *P* = (*F*_o_^2^ + 2*F*_c_^2^)/3
2870 reflections	(Δ/σ)_max_ = 0.014
178 parameters	Δρ_max_ = 0.23 e Å^−^^3^
0 restraints	Δρ_min_ = −0.34 e Å^−^^3^

### Special details

**Table:**

Experimental. CrysAlis RED (Oxford Diffraction, 2009) Empirical absorption correction using spherical harmonics, implemented in SCALE3 ABSPACK scaling algorithm.
Geometry. All esds (except the esd in the dihedral angle between two l.s. planes) are estimated using the full covariance matrix. The cell esds are taken into account individually in the estimation of esds in distances, angles and torsion angles; correlations between esds in cell parameters are only used when they are defined by crystal symmetry. An approximate (isotropic) treatment of cell esds is used for estimating esds involving l.s. planes.
Refinement. Refinement of *F*^2^ against ALL reflections. The weighted *R*-factor wR and goodness of fit *S* are based on *F*^2^, conventional *R*-factors *R* are based on *F*, with *F* set to zero for negative *F*^2^. The threshold expression of *F*^2^ > σ(*F*^2^) is used only for calculating *R*-factors(gt) etc. and is not relevant to the choice of reflections for refinement. *R*-factors based on *F*^2^ are statistically about twice as large as those based on *F*, and *R*- factors based on ALL data will be even larger.

### Fractional atomic coordinates and isotropic or equivalent isotropic displacement parameters (Å^2^)

**Table:**

	*x*	*y*	*z*	*U*_iso_*/*U*_eq_	
C1	0.4436 (2)	0.60399 (12)	0.09959 (19)	0.0371 (4)	
C2	0.4722 (2)	0.55232 (13)	0.2140 (2)	0.0411 (4)	
C3	0.3789 (2)	0.48048 (14)	0.2118 (2)	0.0492 (5)	
H3	0.3962	0.4450	0.2863	0.059*	
C4	0.2619 (2)	0.45894 (14)	0.1049 (3)	0.0522 (6)	
C5	0.2385 (3)	0.51066 (16)	−0.0060 (2)	0.0567 (6)	
H5	0.1616	0.4968	−0.0797	0.068*	
C6	0.3279 (2)	0.58303 (15)	−0.0092 (2)	0.0491 (5)	
H6	0.3103	0.6177	−0.0846	0.059*	
C7	0.3096 (2)	0.79578 (12)	0.12108 (18)	0.0361 (4)	
C8	0.2602 (2)	0.84940 (13)	0.01270 (19)	0.0415 (4)	
H8	0.3302	0.8725	−0.0298	0.050*	
C9	0.1078 (2)	0.86943 (14)	−0.0339 (2)	0.0485 (5)	
C10	0.0062 (3)	0.83549 (16)	0.0326 (3)	0.0574 (6)	
H10	−0.0963	0.8480	0.0027	0.069*	
C11	0.0547 (3)	0.78367 (17)	0.1417 (3)	0.0579 (6)	
H11	−0.0150	0.7622	0.1858	0.069*	
C12	0.2064 (2)	0.76310 (15)	0.1869 (2)	0.0470 (5)	
H12	0.2388	0.7277	0.2607	0.056*	
C13	0.5968 (3)	0.56894 (17)	0.3359 (2)	0.0578 (6)	
H13A	0.5926	0.6284	0.3629	0.069*	
H13B	0.6929	0.5578	0.3168	0.069*	
H13C	0.5841	0.5312	0.4058	0.069*	
C14	0.1627 (3)	0.38099 (18)	0.1097 (3)	0.0747 (8)	
H14A	0.1046	0.3909	0.1738	0.090*	
H14B	0.2248	0.3303	0.1341	0.090*	
H14C	0.0955	0.3722	0.0243	0.090*	
C15	0.0547 (3)	0.92755 (19)	−0.1527 (3)	0.0722 (8)	
H15A	0.1317	0.9310	−0.2010	0.087*	
H15B	0.0345	0.9847	−0.1239	0.087*	
H15C	−0.0361	0.9040	−0.2087	0.087*	
N1	0.46833 (19)	0.77649 (11)	0.16714 (16)	0.0385 (4)	
H1N	0.499 (3)	0.7705 (15)	0.249 (2)	0.046*	
O1	0.69751 (16)	0.69397 (11)	0.16345 (16)	0.0528 (4)	
O2	0.51059 (18)	0.72673 (10)	−0.04494 (14)	0.0497 (4)	
S1	0.54326 (5)	0.70137 (3)	0.09104 (5)	0.03742 (15)	

### Atomic displacement parameters (Å^2^)

**Table:**

	*U*^11^	*U*^22^	*U*^33^	*U*^12^	*U*^13^	*U*^23^
C1	0.0366 (9)	0.0369 (10)	0.0394 (10)	0.0039 (8)	0.0121 (8)	−0.0016 (8)
C2	0.0402 (10)	0.0411 (11)	0.0437 (11)	0.0093 (8)	0.0132 (8)	0.0034 (8)
C3	0.0516 (12)	0.0411 (11)	0.0600 (13)	0.0067 (9)	0.0230 (10)	0.0058 (10)
C4	0.0462 (12)	0.0414 (11)	0.0754 (16)	−0.0002 (9)	0.0270 (11)	−0.0091 (11)
C5	0.0492 (13)	0.0552 (14)	0.0613 (14)	−0.0057 (11)	0.0049 (10)	−0.0138 (11)
C6	0.0516 (12)	0.0511 (13)	0.0411 (11)	0.0000 (10)	0.0044 (9)	−0.0020 (9)
C7	0.0386 (10)	0.0353 (10)	0.0351 (9)	−0.0017 (8)	0.0106 (7)	−0.0069 (8)
C8	0.0456 (11)	0.0397 (10)	0.0417 (10)	−0.0010 (9)	0.0154 (8)	−0.0017 (8)
C9	0.0483 (12)	0.0447 (12)	0.0512 (12)	0.0073 (9)	0.0093 (9)	−0.0012 (9)
C10	0.0404 (12)	0.0573 (14)	0.0746 (16)	0.0049 (10)	0.0141 (11)	−0.0018 (12)
C11	0.0488 (13)	0.0625 (15)	0.0695 (15)	−0.0050 (11)	0.0283 (11)	0.0010 (12)
C12	0.0501 (12)	0.0504 (12)	0.0434 (11)	−0.0025 (10)	0.0167 (9)	0.0015 (9)
C13	0.0599 (14)	0.0602 (14)	0.0476 (13)	0.0055 (12)	0.0021 (10)	0.0126 (11)
C14	0.0625 (16)	0.0534 (15)	0.116 (2)	−0.0128 (12)	0.0367 (16)	−0.0095 (15)
C15	0.0686 (17)	0.0737 (18)	0.0720 (17)	0.0219 (14)	0.0124 (13)	0.0188 (14)
N1	0.0411 (9)	0.0435 (9)	0.0297 (8)	−0.0028 (7)	0.0064 (7)	−0.0042 (7)
O1	0.0348 (8)	0.0641 (10)	0.0587 (9)	−0.0024 (7)	0.0101 (7)	0.0024 (8)
O2	0.0614 (9)	0.0565 (9)	0.0359 (7)	−0.0010 (7)	0.0207 (7)	0.0015 (7)
S1	0.0361 (3)	0.0441 (3)	0.0336 (3)	−0.0008 (2)	0.01146 (18)	0.0008 (2)

### Geometric parameters (Å, °)

**Table:**

C1—C6	1.386 (3)	C10—C11	1.371 (4)
C1—C2	1.403 (3)	C10—H10	0.9300
C1—S1	1.764 (2)	C11—C12	1.385 (3)
C2—C3	1.390 (3)	C11—H11	0.9300
C2—C13	1.506 (3)	C12—H12	0.9300
C3—C4	1.383 (3)	C13—H13A	0.9600
C3—H3	0.9300	C13—H13B	0.9600
C4—C5	1.376 (3)	C13—H13C	0.9600
C4—C14	1.509 (3)	C14—H14A	0.9600
C5—C6	1.383 (3)	C14—H14B	0.9600
C5—H5	0.9300	C14—H14C	0.9600
C6—H6	0.9300	C15—H15A	0.9600
C7—C8	1.382 (3)	C15—H15B	0.9600
C7—C12	1.386 (3)	C15—H15C	0.9600
C7—N1	1.439 (2)	N1—S1	1.6402 (17)
C8—C9	1.390 (3)	N1—H1N	0.83 (2)
C8—H8	0.9300	O1—S1	1.4291 (15)
C9—C10	1.385 (3)	O2—S1	1.4298 (15)
C9—C15	1.508 (3)		
			
C6—C1—C2	120.74 (19)	C12—C11—H11	119.7
C6—C1—S1	117.03 (16)	C11—C12—C7	119.4 (2)
C2—C1—S1	122.10 (15)	C11—C12—H12	120.3
C3—C2—C1	116.67 (19)	C7—C12—H12	120.3
C3—C2—C13	118.75 (19)	C2—C13—H13A	109.5
C1—C2—C13	124.57 (19)	C2—C13—H13B	109.5
C4—C3—C2	123.5 (2)	H13A—C13—H13B	109.5
C4—C3—H3	118.2	C2—C13—H13C	109.5
C2—C3—H3	118.2	H13A—C13—H13C	109.5
C5—C4—C3	118.1 (2)	H13B—C13—H13C	109.5
C5—C4—C14	121.0 (2)	C4—C14—H14A	109.5
C3—C4—C14	120.9 (2)	C4—C14—H14B	109.5
C4—C5—C6	120.8 (2)	H14A—C14—H14B	109.5
C4—C5—H5	119.6	C4—C14—H14C	109.5
C6—C5—H5	119.6	H14A—C14—H14C	109.5
C5—C6—C1	120.1 (2)	H14B—C14—H14C	109.5
C5—C6—H6	119.9	C9—C15—H15A	109.5
C1—C6—H6	119.9	C9—C15—H15B	109.5
C8—C7—C12	119.67 (19)	H15A—C15—H15B	109.5
C8—C7—N1	119.79 (17)	C9—C15—H15C	109.5
C12—C7—N1	120.51 (18)	H15A—C15—H15C	109.5
C7—C8—C9	121.15 (19)	H15B—C15—H15C	109.5
C7—C8—H8	119.4	C7—N1—S1	119.08 (13)
C9—C8—H8	119.4	C7—N1—H1N	115.2 (16)
C10—C9—C8	118.3 (2)	S1—N1—H1N	109.8 (16)
C10—C9—C15	120.9 (2)	O1—S1—O2	119.10 (9)
C8—C9—C15	120.8 (2)	O1—S1—N1	105.78 (9)
C11—C10—C9	120.9 (2)	O2—S1—N1	106.05 (9)
C11—C10—H10	119.5	O1—S1—C1	110.99 (9)
C9—C10—H10	119.5	O2—S1—C1	107.19 (9)
C10—C11—C12	120.6 (2)	N1—S1—C1	107.04 (9)
C10—C11—H11	119.7		
			
C6—C1—C2—C3	−0.2 (3)	C8—C9—C10—C11	0.2 (4)
S1—C1—C2—C3	175.52 (14)	C15—C9—C10—C11	−179.0 (2)
C6—C1—C2—C13	178.9 (2)	C9—C10—C11—C12	−0.9 (4)
S1—C1—C2—C13	−5.4 (3)	C10—C11—C12—C7	0.3 (4)
C1—C2—C3—C4	−0.5 (3)	C8—C7—C12—C11	1.0 (3)
C13—C2—C3—C4	−179.7 (2)	N1—C7—C12—C11	179.05 (19)
C2—C3—C4—C5	1.2 (3)	C8—C7—N1—S1	−80.5 (2)
C2—C3—C4—C14	−178.8 (2)	C12—C7—N1—S1	101.5 (2)
C3—C4—C5—C6	−1.2 (3)	C7—N1—S1—O1	−176.76 (14)
C14—C4—C5—C6	178.8 (2)	C7—N1—S1—O2	55.85 (17)
C4—C5—C6—C1	0.5 (4)	C7—N1—S1—C1	−58.35 (17)
C2—C1—C6—C5	0.2 (3)	C6—C1—S1—O1	−148.09 (16)
S1—C1—C6—C5	−175.76 (17)	C2—C1—S1—O1	36.00 (19)
C12—C7—C8—C9	−1.7 (3)	C6—C1—S1—O2	−16.48 (19)
N1—C7—C8—C9	−179.83 (18)	C2—C1—S1—O2	167.61 (15)
C7—C8—C9—C10	1.1 (3)	C6—C1—S1—N1	96.94 (17)
C7—C8—C9—C15	−179.6 (2)	C2—C1—S1—N1	−78.97 (17)

### Hydrogen-bond geometry (Å, °)

**Table:**

*D*—H···*A*	*D*—H	H···*A*	*D*···*A*	*D*—H···*A*
N1—H1N···O2^i^	0.83 (2)	2.13 (3)	2.932 (2)	162 (2)

Symmetry codes: (i) *x*, −*y*+3/2, *z*+1/2.
